# Supplementary material for: Patient preferences for HIV service delivery models; a Discrete Choice Experiment in Kisumu, Kenya
Source: PLOS Glob Public Health. 2022 Oct 27;2(10):e0000614. doi: 10.1371/journal.pgph.0000614 (PMC10021384; doi:10.1371/journal.pgph.0000614)
Supplement: S1 Text — (DOCX) [file pgph.0000614.s011.docx]

S11 Text: Attribute Importance and Part Worth Utilities

To assess the importance of each attribute, we consider how much difference each attribute makes in the total utility of the preferences. This difference is produced by subtracting the smallest attribute level utility value from the largest attribute level values. The percentage importance is computed by generating proportions from ranges that will just add to 100 percent.

To compute the importance of an attribute, we calculated the difference in the coefficients of the highest and the lowest attribute levels in each attribute. For example, to compute the importance of attitude of care providers e.g., good attitude and bad attitude, if half of the respondents preferred good attitude, then the average utility of good and bad attitude would be tied, and the importance of attitude of care providers is zero. The measures of importance are ratio-scaled – in that a 10% preference is twice as much as 5% preference- and at the same time relative -to the other attributes, study specific [1]. For instance, if the percentage importance of a person providing ART refill is 20% and for attitude of care providers is 10% then the importance of person providing ART refill is twice as much as that of the attitude of care providers [1]

Latent Class Analysis

To choose the most informative number of classes, we used the Akaike’s Information Criterion (AIC), Best Information Criterion (BIC) and consistent AIC (CAIC). The smaller values are considered the better. Class membership was predicted using Multinomial (polytomous) logistic regression, report relative-risk ratios (rrr). The significant p-values qualify the attribute levels to the class, while the **β** indicates the level of preference in the class**.**

Reference

1. Orme BK. Getting Started with Conjoint Analysis: Strategies for Product Design and Pricing Research. Research Publishers LLC; 2014. (Business/Economics/Science).
