# Supplementary material for: Patient preferences for HIV service delivery models; a Discrete Choice Experiment in Kisumu, Kenya
Source: PLOS Glob Public Health. 2022 Oct 27;2(10):e0000614. doi: 10.1371/journal.pgph.0000614 (PMC10021384; doi:10.1371/journal.pgph.0000614)
Supplement: Attachment — Submitted filename: Response to Reviewers.docx [file pgph.0000614.s013.docx]

PGPH-D-21-01122
Patient preferences for HIV service delivery models; A Discrete Choice Experiment in Kisumu, Kenya
PLOS Global Public Health

**Dear Reviewers,**

Thank you for constructive reviews on our manuscript. We have responded to the review queries and answered the questions on an item-to-item basis and revised the manuscript to meet PLOS Global Public Health’s publication criteria. We are therefore happy to submit a revised version of the manuscript that addresses the points raised during the review process.

Overall, this paper is well-written and represents an important contribution to the growing literature on evidence for HIV treatment delivery models, and this is a unique contribution to incorporating local patient preferences to inform the structure of HIV delivery options in Kenya. The strengths of the manuscript are its topical importance, the use of innovative methods to assess patient preferences, and the thoughtful application of findings to considerations for program design.

***Response: This is well appreciated by all the authors of the manuscript.***

The main weaknesses of the manuscript are that it lacks references for the background, lacks sufficient description in some places of interpretation of findings from methods that will be novel to most readers, and in other places spends too much time on details that may be best allocated to a supplemental appendix or tables. As a result, the paper becomes repetitious and unnecessarily wordy at times. Minor grammatical errors throughout--suggest a final read-over for grammar & punctuation. All concerns are minor and addressable. Below are enumerated comments to address:

***Response: See our response below***

Major comments:
1. Lines 81-110, Background: Important background to set up the motivation for the study; please provide references. No references are cited in the entire background section. (Note -- it looks like you have these references in your reference list, they just never appear enumerated in the background).

***Response: References have been inserted and updated in the references section appropriately.***

2. Line 130: Consider using PLHIV instead of "HIV-positive clients." You go on to use person-first language to describe people with HIV; recommend starting with it here.

***Response: The text was changed to PLHIV from HIV-positive clients and the use of first-person language revised appropriately.***

3. Line 134: What models of DSD are available to clients at these centers? ie how much of a ppt's own experience might inform their choices in the DCE, or were most of the options seen as largely theoretical/ideas that they had not been exposed to in practice?

***Response: The patients had the options to choose from the Differentiated Service Delivery Models are fast track/express, standard care, facility-based ART groups, community-based art groupings and community ART groups. Added at line 147 in the methods section of the manuscript.***

4. Line 583, Table 2: Suggest limiting to 2-3 significant figures for percents & CIs. Also, in the % column, you use decimals but I believe you want to use whole percents. (e.g. Income Below 5000 (unit? Ks/month?) N=129, %=54.4 (not 0.5443).

***Response: Adjusted appropriately to 3 significant figures for percentage and confidence intervals.***

Overall for description of the analytic methods, it might be helpful to make the descriptions more concise. Use references to the methods you use, rather than describing precisely how each method works. If you wish to describe methods more extensively, consider placing a longer description of the methods, model-fitting, and selection process into a supplemental appendix.

***Response: Sections on the attribute importance and part worth utilities and Latent Class Analysis deleted and replaced with references. Deleted text moved as supplemental materials.***

5. Lines 217 - 234 Willingness to Trade Analysis: This section is very wordy and sometimes repetitive. For example, you use the phrase "we fit our final model..." twice. Can you streamline the writing of this paragraph?

***Response: revised appropriately to make it more concise****.*

6. Line 131 - is there a reference for the non-traditional WTA?

***Response-Reference added for the non-traditional willingness to trade analysis.***

***these are -insert; Willingness to pay for complete symptom relief of gastroesophageal reflux disease***

***https://pubmed.ncbi.nlm.nih.gov/12076234/***

***-Understanding preferences for HIV care and treatment in Zambia: Evidence from a discrete choice experiment among patients who have been lost to follow-up.***

***https://pubmed.ncbi.nlm.nih.gov/30102693/***

7. Results: In the text of the results, it is helpful to place the reference to the relevant figure or table as close as possible to the beginning of the paragraph that discusses them (rather than at the end of the paragraph).

***Response: Changed appropriately.***

8. Lines 267 - 278. Can you give some context for the magnitude of the preference weights? You note that the strongest preference was provider attitude; is a beta of 1.61 considered large? Given the confidence intervals overlap between the B for provider attitude and the B for ART refill location, are these in fact significantly different, or is the relative strength of the preference for these two attributes of similar importance? Interpreting this for the reader is probably more important than providing a lengthy explanation of how the model was created.

***Response: The attributes are of near similar importance based on their confidence intervals, however if we assess only by looking at their point prevalence, one is slightly higher. Added an introductory statement on how to interpret the preferences as follows, "A positive stated preference value of above 1 indicate a strong preference, below 1 but above 0.5 indicate a mild preference, while below 0.5 indicate a low preference."***

9. Line 280 - Not clear to the reader how the coefficients presented reflect the willingness to trade? Consider changing the methods to describe what the coefficients mean (how to interpret them) rather than describing at length the models that generate them.

***Response: Statements added for willingness to trade at the end of the paragraph. as follows, "A positive preference value of above 1 indicate a strong preference, below 1 but above 0.5 indicate a mild preference, while below 0.5 indicate a low preference."***

10. Same for LCA results -- what is the significance of the delta-Betas?

***Response: Added a statement on LCA interpretation of results as follows, "The significant p-values qualify the attribute levels to the class, while the β indicates the level of preference in the class."***

11. Discussion: start by framing the discussion with a summary statement of what you did -- "In this study of PLHIV patient preferences for DSD components, respondents had the strongest preference...."

***Response: We changed the introduction for the discussion by starting with, "In this study of PLHIV patient preferences for DSD components, respondents had the strongest preference...."***

12. Line 320. What did the study in Ghana show? Helpful to give a 1-2 sentence summary to demonstrate the consistence or discordance of the results.

***Response: Text added as follows in the manuscript "the study found that "fear of stigma and discrimination was very strong and the main barrier to community-based models..."***

Also, do you find this surprising? In many places where CAGs are used, CAG participants are quite happy to receive refills in the CAG setting outside of the facility. Did participants who were respondents in the DCE have exposure to CAGs? Could it be that the reason that facilities were the preferred refill setting was because there was a lack of familiarity with the other options?

***Response: All the patients had the opportunity to be explored the available options of DSD by regular interaction of with the adherence and retention officers during their routine clinical and drug pick -up visits.*** **It could also be true that our sample has a few respondents who were in CAGs and their views may be under represented.** **Fast-track is also the most preferred model of care.**

**We added the line “**It’s not just important to pay attention to patient preferences but also that our findings were consistent with other studies that patients strongly prefer health facilities over CAG models (for whatever reason) and programs should consider shifting DSDs toward that.**” in the discussions at line 368 -371 to further address this.**

Minor concerns:

1. Line 96: Editing error "[describe an example of two very briefly here]" should be deleted and replaced with description.

***Response: revised appropriately***

2. Line 98: delete "for example;" unnecessarily wordy.

***Response: deleted.***

Line 100: "Instead, the literature needs research..." The literature doesn't need research; national programs, implementers, and funders need research.

***Response: "Instead, the literature needs research..." This has been revised in a different way.***

3. Line 102: 1st sentence - very wordy. Can you shorten to make more concise?

***Response: changed appropriately.***

4. Line 110: please define abbreviations DSD, LMIC in the first instance

***Response: DSD defined in the first instance of the abstract in the background section, LMIC-***

5. Line 113: Please define abbreviation DCE in the first instance in the text, then can use abbreviation after

***Response: defined in the abstract of the document.***

6. Line 158-161. Not clear how multi-month scripting (described as "another component of the care model") is different from reduced frequency of ART pick-ups. Are these in fact different?

***Response: This is the same thing, rephrased from another manuscript referenced here that described the same thing.***

7. Line 168 - consider giving more examples of types of people providing ART refills -- clinician, pharmacy, peer, or self-collect e.g., through vending machine.

**Response: additional text added in the manuscript as suggested**

8. Line 182. Instead of providing Table 1 here in the first instance, suggest providing the Table 1 link at the beginning of this section, so the reader can follow along with the Table as they read more about each attribute/level.

***Response: Figure 1 referencing moved to earlier in the document***

9. Line 198 - avoid using emdashes in writing (e.g. put "expressed as a percentage" in regular text, at the end of the sentence (or in parentheses), or consider eliminating entirely. Same for Line 202 "generated from the attributes."

**Response: the emdashes have been removed. Same for Line 202 "generated from the attributes." removed**

10. Line 202 - is there a reference for this method that you can cite?

***Response: Reference inserted https://sawtoothsoftware.com/resources/technical-papers/interpreting-conjoint-analysis-data***

11. Line 253 "The median age among the respondents was 38 (31-44)." Is 31-44 an IQR? Range? Please specify in text.

***Response: interquartile range -updated appropriately.***

12. Results -- again, limit %s to one digit after decimal place.

***Response: -changed to decimal.***

13. Line 260 - repetitive to say that "about 20%" of respondents selected location of ART refills...and then provide the exact percentage. Just use one percentage (ideally the exact number).

**Response: revised to remove the repetitive nature.**

14. Line 662 - Table 5 - p-values should not be 0.000 anywhere -- if that is the software output, please present as <0.001 (p-values are never truly 0!)

***Response: changed all instances of 0.000 to <0.001.***

15. Line 665 - Are these Incidence Rate Ratios as the table header suggests? or RRR, as the column header suggests?

**Response: relative-risk ratios.**

16. Line 323 – Referencing

***Response: updated to the right format***

17. Line 325 "resource-limited settings" instead of "poor resource settings"

**Response: updated appropriately**

18. Line 354 - Agree with your strengths, but also important to include some limitations. One potential limitation you should address is how generalizable these results are. What do you think is the relevant application for your results for DSD program design? Kenya? Outside of Kenya? You give many examples in the discussion of DSD models in diverse locations such as Ghana, Zimbabwe, and China. Do you think the settings are comparable? As you make your conclusions, important to reflect on the generalizability of your findings. (It's not a flaw of the study if the results only apply to Kenya, or to one region of Kenya, but it should be acknowledged).

**Response: Added a line on limitations before the strengths paragraph as follows, " The main limitation of our investigations is on the generalizability of the findings beyond the care settings in Kenya. It could be also that few of our respondents were in CAGs and so their views may be underrepresented because fast-track is more widely adopted and therefore fewer participants had experience with CAGs."**

Reviewer 2
The methodology appears quite rigorous but may need some improvement particularly the study design which lacks detail. Explain in more detail how the Discrete Choice Experiment (DCE) was conducted. Also, what’s your exclusion criteria?

**Response: We explain in line 196/202 of the tracked document, It reads, “Each respondent received a randomly generated set of…”**

**Our exclusion criteria have been updated as follows in line 143/146 of the tracked document as follows, “The exclusion criteria for the for PLHIV were: not enrolled in HIV care one of the four health facilities, age below 18 years, having been on ART for less than six months, not willing to provide informed consent, and not able to communicate in English, Kiswahili, or the Dholuo language.”**


[Note: HTML markup is below. Please do not edit.]

Response ends,

On behalf of the co-authors

**Raphael Mando Onyango**

Reviewers' comments:

Reviewer's Responses to Questions

**Comments to the Author**

1. Does this manuscript meet PLOS Global Public Health’s [publication criteria](https://nam10.safelinks.protection.outlook.com/?url=https://urldefense.com/v3/__https:/nam10.safelinks.protection.outlook.com/?url%3Dhttps%2a3A%2a2F%2a2Fjournals.plos.org%2a2Fglobalpublichealth%2a2Fs%2a2Fcriteria-for-publication%26data%3D05%2a7C01%2a7Celvin.geng%2a40wustl.edu%2a7Cf101694d5fb3425e14fa08da6a1d06ee%2a7C4ccca3b571cd4e6d974b4d9beb96c6d6%2a7C0%2a7C0%2a7C637938970181147451%2a7CUnknown%2a7CTWFpbGZsb3d8eyJWIjoiMC4wLjAwMDAiLCJQIjoiV2luMzIiLCJBTiI6Ik1haWwiLCJXVCI6Mn0%2a3D%2a7C3000%2a7C%2a7C%2a7C%26sdata%3DBcWOUWiIboLZYyivNRgNVxeno28WjVmtJwaPpFaMffY%2a3D%26reserved%3D0__%3bJSUlJSUlJSUlJSUlJSUlJSUlJSUlJQ%21%21LQC6Cpwp%21thLn4NiS_bgf5i5mqjr1Rd7RSE2_kN68XXkeAn9d3i2JfaOzeUuhP5mb--avdxhVols1sIzuVRE9v6GvQtuqjfw26Qo$&data=05%7c01%7celvin.geng%40wustl.edu%7c4ef877baadee4d78084508da763f26f9%7c4ccca3b571cd4e6d974b4d9beb96c6d6%7c0%7c0%7c637952310877656640%7cUnknown%7cTWFpbGZsb3d8eyJWIjoiMC4wLjAwMDAiLCJQIjoiV2luMzIiLCJBTiI6Ik1haWwiLCJXVCI6Mn0%3D%7c3000%7c%7c%7c&sdata=VpV%2BqDoHPzhp7M%2BlqPGjliGIDWH8TH8LfDNGXztOBBM%3D&reserved=0)? Is the manuscript technically sound, and do the data support the conclusions? The manuscript must describe methodologically and ethically rigorous research with conclusions that are appropriately drawn based on the data presented.

Reviewer #1: Yes

Reviewer #2: Partly

2. Has the statistical analysis been performed appropriately and rigorously?

Reviewer #1: I don't know

Reviewer #2: Yes

3. Have the authors made all data underlying the findings in their manuscript fully available (please refer to the Data Availability Statement at the start of the manuscript PDF file)?

The [PLOS Data policy](https://nam10.safelinks.protection.outlook.com/?url=https://urldefense.com/v3/__https:/nam10.safelinks.protection.outlook.com/?url%3Dhttps%2a3A%2a2F%2a2Fjournals.plos.org%2a2Fglobalpublichealth%2a2Fs%2a2Fdata-availability%26data%3D05%2a7C01%2a7Celvin.geng%2a40wustl.edu%2a7Cf101694d5fb3425e14fa08da6a1d06ee%2a7C4ccca3b571cd4e6d974b4d9beb96c6d6%2a7C0%2a7C0%2a7C637938970181147451%2a7CUnknown%2a7CTWFpbGZsb3d8eyJWIjoiMC4wLjAwMDAiLCJQIjoiV2luMzIiLCJBTiI6Ik1haWwiLCJXVCI6Mn0%2a3D%2a7C3000%2a7C%2a7C%2a7C%26sdata%3D2SR13co7ULO8bJNq8ClGouJ8ecqHflQaa6%2a2BcrW942RU%2a3D%26reserved%3D0__%3bJSUlJSUlJSUlJSUlJSUlJSUlJSUlJSU%21%21LQC6Cpwp%21thLn4NiS_bgf5i5mqjr1Rd7RSE2_kN68XXkeAn9d3i2JfaOzeUuhP5mb--avdxhVols1sIzuVRE9v6GvQtuq0Kts1-s$&data=05%7c01%7celvin.geng%40wustl.edu%7c4ef877baadee4d78084508da763f26f9%7c4ccca3b571cd4e6d974b4d9beb96c6d6%7c0%7c0%7c637952310877656640%7cUnknown%7cTWFpbGZsb3d8eyJWIjoiMC4wLjAwMDAiLCJQIjoiV2luMzIiLCJBTiI6Ik1haWwiLCJXVCI6Mn0%3D%7c3000%7c%7c%7c&sdata=FssCPWK2CTLjQmsqhy68YqEj9AYKMZ400UbjyCDSCis%3D&reserved=0) requires authors to make all data underlying the findings described in their manuscript fully available without restriction, with rare exception. The data should be provided as part of the manuscript or its supporting information, or deposited to a public repository. For example, in addition to summary statistics, the data points behind means, medians and variance measures should be available. If there are restrictions on publicly sharing data—e.g. participant privacy or use of data from a third party—those must be specified.

Reviewer #1: No

Reviewer #2: Yes

4. Is the manuscript presented in an intelligible fashion and written in standard English?

PLOS Global Public Health does not copyedit accepted manuscripts, so the language in submitted articles must be clear, correct, and unambiguous. Any typographical or grammatical errors should be corrected at revision, so please note any specific errors here.

Reviewer #1: Yes

Reviewer #2: Yes

5. Review Comments to the Author

Please use the space provided to explain your answers to the questions above. You may also include additional comments for the author, including concerns about dual publication, research ethics, or publication ethics. (Please upload your review as an attachment if it exceeds 20,000 characters)

Reviewer #1: To the authors:

Thank you for the opportunity to review this manuscript on patient preferences for HIV health care delivery, assessed by a discrete choice experiment. Overall this paper is well-written and represents an important contribution to the growing literature on evidence for HIV treatment delivery models, and this is a unique contribution to incorporating local patient preferences to inform the structure of HIV delivery options in Kenya. The strengths of the manuscript are its topical importance, the use of innovative methods to assess patient preferences, and the thoughtful application of findings to considerations for program design.

The main weaknesses of the manuscript are that it lacks references for the background, lacks sufficient description in some places of interpretation of findings from methods that will be novel to most readers, and in other places spends too much time on details that may be best allocated to a supplemental appendix or tables. As a result, the paper becomes repetitious and unnecessarily wordy at times. Minor grammatical errors throughout--suggest a final read-over for grammar & punctuation. All concerns are minor and addressable. Below are enumerated comments to address:

Major comments:
1. Lines 81-110, Background: Important background to set up the motivation for the study; please provide references. No references are cited in the entire background section. (Note -- it looks like you have these references in your reference list, they just never appear enumerated in the backround).

2. Line 130: Consider using PLHIV instead of "HIV-positive clients." You go on to use person-first language to describe people with HIV; recommend starting with it here.

3. Line 134: What models of DSD are available to clients at these centers? ie how much of a ppt's own experience might inform their choices in the DCE, or were most of the options seen as largely theoretical/ideas that they had not been exposed to in practice?

4. Line 583, Table 2: Suggest limiting to 2-3 significant figures for percents & CIs. Also, in the % column, you use decimals but I believe you want to use whole percents. (e.g. Income Below 5000 (unit? Ks/month?) N=129, %=54.4 (not 0.5443).

Overall for description of the analytic methods, it might be helpful to make the descriptions more concise. Use references to the methods you use, rather than describing precisely how each method works. If you wish to describe methods more extensively, consider placing a longer description of the methods, model-fitting, and selection process into a supplemental appendix.

5. Lines 217 - 234 Willingness to Trade Analysis: This section is very wordy and sometimes repetitive. For example you use the phrase "we fit our final model..." twice. Can you streamline the writing of this paragraph?
6. Line 131 - is there a reference for the non-traditional WTA?

7. Results: In the text of the results, it is helpful to place the reference to the relevant figure or table as close as possible to the beginning of the paragraph that discusses them (rather than at the end of the paragraph).

8. Lines 267 - 278. Can you give some context for the magnitude of the preference weights? You note that the strongest preference was provider attitude; is a beta of 1.61 considered large? Given the confidence intervals overlap between the B for provider attitude and the B for ART refill location, are these in fact significantly different, or is the relative strength of the preference for these two attributes of similar importance? Interpreting this for the reader is probably more important than providing a lengthy explanation of how the model was created.
9. Line 280 - Not clear to the reader how the coeffecients presented reflect the willingness to trade? Consider changing the methods to describe what the coefficients mean (how to interpret them) rather than describing at length the models that generate them.
10. Same for LCA results -- what is the significance of the delta-Betas?

11. Discussion: start by framing the discussion with a summary statement of what you did -- "In this study of PLHIV patient preferences for DSD components, respondents had the strongest preference...."

12. Line 320. What did the study in Ghana show? Helpful to give a 1-2 sentence summary to demonstrate the consistence or discordance of the results.

Also, do you find this surprising? In many places where CAGs are used, CAG participants are quite happy to receive refills in the CAG setting outside of the facility. Did participants who were respondents in the DCE have exposure to CAGs? Could it be that the reason that facilities were the preferred refill setting was because there was a lack of familiarity with the other options?


Line

Minor concerns:

1. Line 96: Editing error "[describe an example of two very briefly here]" should be deleted and replaced with description.
2. Line 98: delete "for example;" unnecessarily wordy.
Line 100: "Instead, the literature needs research..." The literature doesn't need research; national programs, implementers, and funders need research.
3. Line 102: 1st sentence - very wordy. Can you shorten to make more concise?
4. Line 110: please define abbreviations DSD, LMIC in the first instance
5. Line 113: Please define abbreviation DCE in the first instance in the text, then can use abbreviation after
6. Line 158-161. Not clear how multi-month scripting (described as "another component of the care model") is different from reduced frequency of ART pick-ups. Are these in fact different?
7. Line 168 - consider giving more examples of types of people providing ART refills -- clinician, pharmacy, peer, or self-collect e.g. through vending machine.
8. Line 182. Instead of providing Table 1 here in the first instance, suggest providing the Table 1 link at the beginning of this section, so the reader can follow along with the Table as they read more about each attribute/level.
9. Line 198 - avoid using emdashes in writing (e.g. put "expressed as a percentage" in regular text, at the end of the sentence (or in parentheses), or consider eliminating entirely. Same for Line 202 "generated from the attributes."
10. Line 202 - is there a reference for this method that you can cite?
11. Line 253 "The median age among the respondents was 38 (31-44)." Is 31-44 an IQR? Range? Please specify in text.
12. Results -- again, limit %s to one digit after decimal place.
13. Line 260 - repetitive to say that "about 20%" of respondents selected location of ART refills...and then provide the exact percentage. Just use one percentage (ideally the exact number).
14. Line 662 - Table 5 - p-values should not be 0.000 anywhere -- if that is the software output, please present as <0.001 (p-values are never truly 0!)
15. Line 665 - Are these Incidence Rate Ratios as the table header suggests? or RRR, as the column header suggests?
16. Line 323 - Referencing
17. Line 325 "resource-limited settings" instead of "poor resource settings"
18. Line 354 - Agree with your strengths, but also important to include some limitations. One potential limitation you should address is how generalizable these results are. What do you think is the relevant application for your results for DSD program design? Kenya? Outside of Kenya? You give many examples in the discussion of DSD models in diverse locations such as Ghana, Zimbabwe, and China. Do you think the settings are comparable? As you make your conclusions, important to reflect on the generalizability of your findings. (It's not a flaw of the study if the results only apply to Kenya, or to one region of Kenya, but it should be acknowledged).

Reviewer #2: The methodology appears quite rigorous but may need some improvement particularly the study design which lacks detail. Explain in more detail how the Discrete Choice Experiment(DCE) was conducted . Also What's your exclusion criteria?

Please submit your revised manuscript by Aug 12 2022 11:59PM. If you will need more time than this to complete your revisions, please reply to this message or contact the journal office at globalpubhealth@plos.org. When you're ready to submit your revision, log on to [https://www.editorialmanager.com/pgph/](https://nam10.safelinks.protection.outlook.com/?url=https://urldefense.com/v3/__https:/www.editorialmanager.com/pgph/__%3b%21%21LQC6Cpwp%21thLn4NiS_bgf5i5mqjr1Rd7RSE2_kN68XXkeAn9d3i2JfaOzeUuhP5mb--avdxhVols1sIzuVRE9v6GvQtuqHxfydfM$&data=05%7c01%7celvin.geng%40wustl.edu%7c4ef877baadee4d78084508da763f26f9%7c4ccca3b571cd4e6d974b4d9beb96c6d6%7c0%7c0%7c637952310877656640%7cUnknown%7cTWFpbGZsb3d8eyJWIjoiMC4wLjAwMDAiLCJQIjoiV2luMzIiLCJBTiI6Ik1haWwiLCJXVCI6Mn0%3D%7c3000%7c%7c%7c&sdata=UPqxljxV4ihndaAafQhM311UJvq3oq/rUVa/BDK9xpU%3D&reserved=0) and select the 'Submissions Needing Revision' folder to locate your manuscript file.

Please include the following items when submitting your revised manuscript:
